# Risk factors for postoperative recurrent tricuspid regurgitation after concomitant tricuspid annuloplasty during left heart surgery and the association between tricuspid annular circumference and secondary tricuspid regurgitation

**DOI:** 10.1186/s12872-021-01870-5

**Published:** 2021-01-26

**Authors:** JinGuo Xu, Jie Han, Haibo Zhang, Fei Meng, Tiange Luo, BaiYu Tian, JianGang Wang, YuQing Jiao, HuiMei Yu, Xu Meng

**Affiliations:** 1 Department of Cardiac Surgery, Beijing Anzhen Hospital, Capital Medical University, No. 2 Anzhen Road, Chaoyang District, Beijing, 100029 China; 2 Echocardiography Division No. 3 Beijing Anzhen Hospital, Capital Medical University, Beijing, 100029 China

**Keywords:** Tricuspid regurgitation, Tricuspid annuloplasty, Tricuspid annular circumference

## Abstract

**Background:**

To identify the association between tricuspid annular circumference and secondary tricuspid regurgitation and analyze the risk factors of recurrent tricuspid regurgitation after concomitant tricuspid annuloplasty during left heart surgery.

**Methods:**

From October 2018 to June 2019, a total of 117 patients receiving concomitant tricuspid annuloplasty within left heart surgery were enrolled. Severity of tricuspid regurgitation was classified as 4 subtypes: normal, mild, moderate and severe. Perioperative data and mid-term outcome were collected. Tricuspid annular circumference (TAC) was measured under cardiac arrest during surgery procedure by cardioplegia. Optimal TAC and TAC index (TAC/body surface area, BSA) cutoffs of significant tricuspid annulus dilatation (moderate and severe) were obtained. Univariable and multivariable logistic regression analyses were performed to identify the risk factors of postoperative recurrent tricuspid regurgitation. The follow up period is 13–19 months (mean 15.5 ± 3.2 months).

**Results:**

There was 1 patient was excluded who died after surgery. A total of 116 patients receiving tricuspid annuloplasty were included. Optimal cutoffs of significant tricuspid annulus dilatation were recommended (TAC 11.45 cm, Sensitivity 82.89%, Specificity 73.68%, AUC 0.915; TAC index 7.09 cm/m^2^, Sensitivity 73.68%, Specificity 85%, AUC 0.825, respectively). Based on findings of multivariable logistic regression, it has been showed that TAC index and postoperative atrial fibrillation were the independent risk factors of recurrent regurgitation after surgery. Optimal TAC index cutoff to predict recurrent tricuspid regurgitation was 7.86 cm/m^2^

**Conclusions:**

The severity of secondary tricuspid regurgitation is associated with the tricuspid annular circumference. The cut-offs of significant tricuspid regurgitation (more than moderate) were TAC 11.45 cm and TAC index 7.09 cm/m^2^, respectively. Clinically, concomitant tricuspid annuloplasty is relative safe and effective. TAC index ≥ 7.86 cm/m^2^ and postoperative atrial fibrillation are the risk factors of recurrent significant tricuspid regurgitation after concomitant tricuspid annuloplasty during left heart surgery.

## Background

Secondary tricuspid regurgitation, known as functional tricuspid regurgitation (FTR), is a kind of common tricuspid disfunction mainly caused by left heart diseases. It has been found that both degenerative mitral insufficiency and rheumatic mitral stenosis are associated with high prevalence of tricuspid regurgitation prevalence. Additionally, about 16% patients with aortic stenosis also suffer moderate or severe tricuspid regurgitation [1]. Preoperatively and postoperatively, right heart function is persistently damaged by serious FTR and also, the incidence of adverse cardiovascular events increases dramatically. However severity of FTR is greatly varied due to diuretics [2], therefore, it is difficult to assess the exact FTR related regurgitation. Previously, it was considered that self-limited FTR would be improved once left heart disease was cured and so mild tricuspid regurgitation was tolerant and without special interventions. However, recently, according to a series of evidence from studies, it has been confirmed that the prevalence of severe tricuspid regurgitation is still high even after left heart surgery, besides untreated tricuspid valve, physiological alternations from treated tricuspid valve should not be ignored [3]. Late severe tricuspid regurgitation often leads to poor outcome and high mortality [4]. Therefore, it is particularly important to accurately assess preoperative severity of tricuspid regurgitation and predict postoperative outcome. The purpose of this study aims to investigate the risk factors of recurrent tricuspid regurgitation after tricuspid annuloplasty, and the association between severity of tricuspid regurgitation and TAC.

## Material and methods

A total of 117 patients with secondary tricuspid regurgitation receiving tricuspid annuloplasty from October 2018 to June 2019 in our medical center. Surgical procedures of left heart include mitral valve repair, mitral valve replacement, mitral valve repair combined with aortic valve replacement, mitral valve replacement combined with aortic valve replacement and atrial septal defect (ASD) closure. Patients with atrial fibrillation (Af) and coronary heart disease receiving simultaneous Cox-MAZE IV and coronary artery bypass grafting, respectively. Patients with right coronary artery disease were excluded.

### Echocardiography

Data of all patients was recorded including preoperative transthoracic echocardiography, tricuspid regurgitation jet area, tricuspid annular plane systolic excursion (TAPSE), left ventricular ejection fraction (LVEF), heart chamber size, pulmonary artery pressure, etc. Tricuspid regurgitation was classified as 4 subtypes based on the ratio of TR jet area/right atrium (RA) area [5]: normal, mild (< 0.2), moderate (0.2–0.33) and severe (> 0.33). All patients were followed up with TTE periodically. The primary end-point was the recurrence of significant tricuspid regurgitation (defined as moderate and severe regurgitation).

### Surgical procedure

**Fig. 1:**
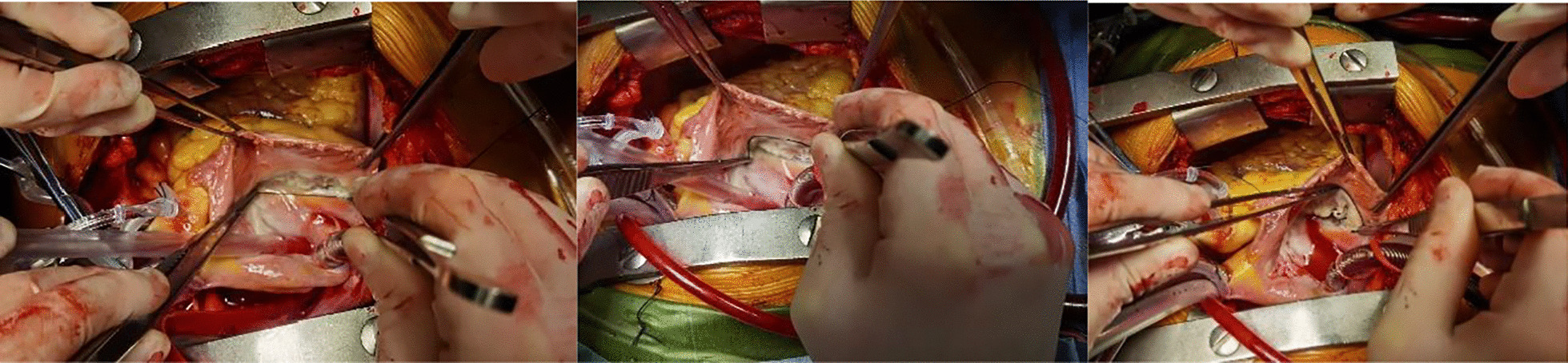
Measurement of tricuspid annulus: **a** measurement of septal annulus; **b** measurement of anterior annulus; **c** measurement of posterior annulus

The median sternotomy was selected for all patients. The cardiopulmonary bypass was done regularly, artery tube for ascending aorta and vein tubes for both superior and inferior vena cava. Myocardial protection was performed through antegrade perfusion of cold blood cardioplegia. Mitral valve was exposed through left interatrial groove pathway and tricuspid valve was exposed through right atrium pathway. The measurement of tricuspid annular circumference must be emphasized. Retractor was gently pulled to expose entire structure of tricuspid valve under cardiac arrest. We have tried to gently lift the atrial wall with two forceps or retractors to expose the entire tricuspid annulus instead of strenuous retraction with the hooks leading to deformation of the annulus and measurement variability. Taken special nonlinear shape of tricuspid valve annulus into consideration, instead of direct measurement, a soft silk line was used to measure indirectly and independently each margin of tricuspid annulus, and then a ruler was used to measure length of the silk line to obtain final size of corresponding margin, after triple attempts, three lengths were combined to calculate tricuspid annular circumference. The photos of the measurement are presented in Fig. 1a–c. Indications of tricuspid annuloplasty were shown as the presence of moderate or severe FTR, significant annular dilation (annulus > 40 mm or 21 mm/m^2^ based on measurement under 4-chamber view), positive intraoperative check confirming the necessity of annuloplasty. Medtronic Contour 3D ring (Medtronic, Minneapolis, Minnesota, USA) and Edwards MC3 ring (Edwards LifeScience, Irvine, CA, USA) were used for tricuspid annuloplasty. Intraannular interrupted suture was generally initiated from center of septal annulus, along with right side of septal, posterior, and anterior annulus, and finally, completed at targeted location, 3–5 mm above anterior-septal commissure. The number of suture was usually 8–10 stitches. All of the patients were rechecked under transesophageal echocardiography to evaluate heart structural improvement after cardiac rehabilitation.

### Statistical analysis

Continuous variables are expressed as the mean ± standard deviation and were analyzed by *t* test. Categorical variables are expressed as percentages and were analyzed by using the chi-square test. Risk factors of recurrence of significant tricuspid regurgitation were analyzed by binary logistic regression. In univariate analysis, significant factors with *P* < 0.05 were included in multivariate analysis, forward conditional logistic regression was used to identify risk factors. The optimal cut-off of the TAC/TACI to assess tricuspid annulus dilatation and validation of predictor of recurrent regurgitation were presented as receiver operating characteristic (ROC) curves. A value of *p* < 0.05 was considered as statistically significance. SPSS 15.0 software (SPSS Inc, Chicago, IL) was used for statistical analysis.

## Results

There was 1 patient died due to pneumonia for 12 days after surgery and was excluded. There were 116 patients were involved with the average age of 57 ± 7.6 years, of whom, the number of male patients was 59. All of the patients were followed up periodically. There was no death case found during follow up with the mean period of 15.5 ± 3.2 months. There were 40 patients diagnosed as normal or mild FTR and 49 patients for moderate FTR as well as 27 patients for severe FTR. Procedures of left heart surgery included mitral valve repair (n = 91), mitral valve repair combined with aortic valve replacement (n = 6), mitral valve replacement (n = 10), mitral and aortic valve replacement (n = 6) and ASD closure (n = 3). The mean cardiopulmonary bypass time was 135 ± 45 min. The mean aorta cross clamp time was 102 ± 39 min. A total of 77 (66.4%) patients suffered with AF were performed simultaneous Cox-MAZE IV procedure, of whom, the conversion of sinus rhythm was found in 66 patients during follow up. A total of 19 (16.4%) patients received coronary artery bypass grafting simultaneously. There was no recurrence of moderate mitral regurgitation and stenosis. Only 1 patient (0.086%) underwent postoperative pacemaker implantation due to high grade atrioventricular conduction block. The number of patients suffering recurrent moderate regurgitation during follow up (longer than 12 months) was 15 (12.9%). The percentage of patient without recurrent tricuspid regurgitation after annuloplasty was 87.1%. Baseline characteristics of patients is shown in Table 1. Operation information data is shown in Table 2.

**Table 1:** Baseline characteristics and echocardiographic data

Variables	No (%)
Male	59 (50.86%)
Age (years)	57 ± 7.6
BSA	1.71 ± 0.19m2
Atrial fibrillation	77
Tricuspid regurgitation	
Normal and mild	40
Moderate	49
Severe	27
RA dimension	44 ± 6.2 mm
RV dimension	36.3 ± 4.8 mm
LA dimension	47.9 ± 7.2 mm
LVEDD	50.7 ± 7.3 mm
LVESD	33.9 ± 5.4 mm
LVEF	0.61 ± 0.05
TAPSE	1.78 ± 0.17 cm
PASP	37.9 ± 12.8 mmHg
TAD	34.3 ± 3.3 mm
NYHA	
Class I	14
Class II	61
Class III	35
Class IV	6

*BSA* body surface area, *RA* right atrium, *RV* right ventricle, *LA* left atrium, *LVEDD* left ventricular end‐diastolic diameter, *LVESD* left ventricular end‐systolic diameter, *LVEF* left ventricular ejection fraction, *TAPSE* tricuspid annular plane systolic excursion, *PASP* pulmonary arterial systolic pressure, *TAD* tricuspid annular dimension in apical four-chamber view, *NYHA* New York Heart Association

**Table 2:** Operation information data

Variables	No (%)
Left-heart surgery	
MVP	91
MVR	10
MVP + AVR	6
MVR + AVR	6
ASD repair	3
Ring for tricuspid valvuloplasty	
Edwards MC3 ring	28# (n = 26) 30# (n = 28) 32# (n = 3)
Medtronic contour 3D ring	28# (n = 32) 30# (n = 23) 32# (n = 4)
Tricuspid annular circumference	11.8 ± 1.3 cm
length of anterior annulus	4.6 ± 0.6 cm
length of septal annulus	3.8 ± 0.4 cm
length of posterior annulus	3.5 ± 0.5 cm
Cox-Maze IV procedure	77
CABG	19

*MVP* mitral vavle repair, *MVR* mitral vavle replacement, *AVR* aortic valve replacement, *ASD* atrial septal defect, *CABG* coronary artery bypass grafting

### Assessment of tricuspid annulus dilatation

**Fig. 2:**
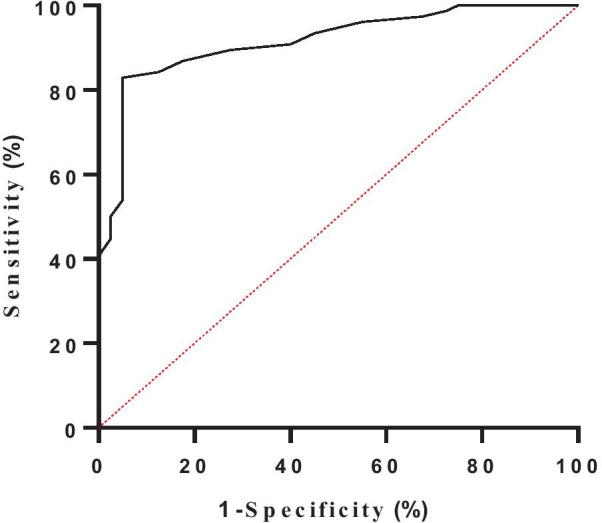
TAC of Receiver-operator curve (ROC) for determining cut-off values to assess tricuspid annulus dilatation

**Fig. 3:**
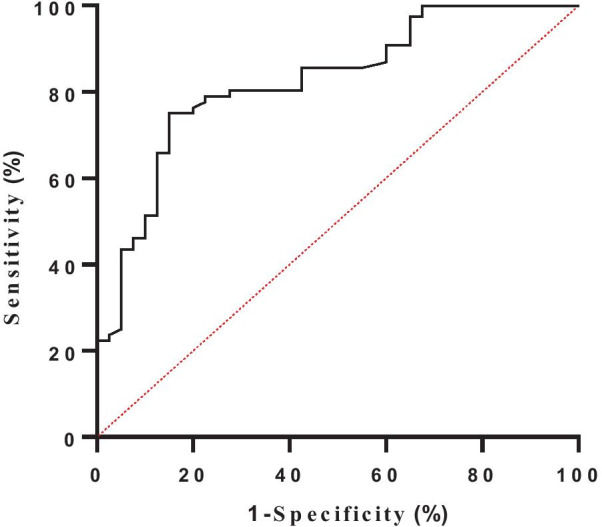
TACI of Receiver-operator curve (ROC) for determining cut-off values to assess tricuspid annulus dilatation

TAC and TACI (TAC index) are shown in Table 3. In order to assess tricuspid annulus dilatation, patients were divided into two groups according to the severity of FTR, group 1 (No significant tricuspid annulus dilatation): normal and mild regurgitation, group 2 (Significant tricuspid annulus dilatation): moderate and severe regurgitation. We found that both TAC and TACI were lager in group 2. Optimal cutoffs were obtained through receiver operating characteristic (ROC) curves analysis to confirm the threshold of annulus dilatation. The TAC with 11.45 cm and TACI with 7.07 cm/m^2^ were considered as the optimal cutoffs defining significant tricuspid annulus dilatation (Table 4 and Figs. 2, 3).

**Table 3:** Tricuspid Annulus circumference obtained by intraoperative measurement

Variables	None significant (n = 40)	Significant (n = 76)	*t* value	*P* value
TAC	10.78 ± 0.70 cm	12.38 ± 1.13 cm	− 9.464	< 0.001
TACI	6.25 ± 0.87 cm/m^2^	7.37 ± 0.80 cm/m^2^	− 7.091	< 0.001

*TAC* tricuspid annular circumference, *TACI* tricuspid annular circumference/body surface area

**Table 4:** Sensitivity and specificity of TAC and TACI for evaluation of significant tricuspid annulus dilatation

Variables	Cut off point	Sensitivity %	Specificity %	AUC	*P* value
TAC	11.45 cm	82.9	95.0	0.915	< 0.001
TACI	7.07 cm/m^2^	75.0	85.0	0.825	< 0.001

*TAC* tricuspid annular circumference, *TACI* tricuspid annular circumference/body surface area

Then the cutoffs (11.45 cm and 7.07 cm/m^2^) were used in group 1 for tricuspid annulus evaluation and there were 2 (under cutoff of 11.45 cm) and 6 (under cutoff of 7.07 cm/m^2^) significant tricuspid annulus dilatation patients, respectively.

### Analysis of risk factors of recurrent regurgitation

During follow up longer than 1 year, 15 patients with the significant recurrent tricuspid regurgitation (moderate and severe tricuspid regurgitation) were found, among whom, except for necessary anticoagulation treatment of warfarin and aspirin, no any other medicine was administrated including diuretics.

### Univariable analysis of risk factors of significant recurrent tricuspid regurgitation

It was demonstrated by univariable analysis that age, height, weight, BSA, LVEF, length of septal annulus, length of posterior annulus, length of anterior annulus, TAC, TACI, gender, preoperative AF, severity of preoperative FTR and postoperative AF were associated with recurrent tricuspid regurgitation (Table 5).

**Table 5:** Univariable analysis of risk factors of significant recurrent tricuspid regurgitation

Variables	Obvious recurrent regurgitation (%)		None obvious recurrent regurgitation (%)		x^2^/t	*P*
Gender						
Male	4	6.8	55	93.2	3.755	0.054
Female	11	19.3	46	80.7		
Left side surgery						
None-MVR	13	13.0	87	87.0	0.003	0.956
MVR	2	12.5	14	87.5		
NYHA class						
1	0	0	14	100	6.155	0.104
2	7	11.5	54	88.5		
3	6	17.1	29	82.9		
4	2	33.3	4	66.7		
Preoperative Af						
Yes	14	18.2	63	81.8	5.608	0.018
No	1	2.6	38	97.4		
Severity of FTR Preoperatively						
Mild	0	0	40	100	18.353	0.001
Moderate	6	12.2	43	87.8		
Severe	9	31.0	18	69.0		
Severity of FTR when discharge						
None	4	8.5	43	91.5	3.284	0.167
Mild	4	10.5	34	89.5		
Moderate	7	22.6	24	77.4		
Postoperative Af						
Yes	7	63.6	4	36.4	27.751	0.001
No	8	7.6	97	92.4		
Age (years)	56.06 ± 11.58		64.33 ± 10.78		2.603	0.010*
BSA (m^2^)	1.73 ± 0.19		1.58 ± 0.13		− 4.156	0.001**
TAPSE (cm)	1.78 ± 0.17		1.73 ± 0.18		− 1.059	0.292
PASP (mmHg)	37.48 ± 13.05		40.53 ± 11.13		0.862	0.391
LA (mm)	47.57 ± 7.02		49.80 ± 8.14		1.116	0.267
RA (mm)	43.79 ± 5.57		45.47 ± 9.63		0.657	0.521
RV (mm)	36.38 ± 4.92		35.87 ± 3.83		− 0.384	0.702
LVEDD (mm)	50.92 ± 7.50		49.40 ± 5.89		− 0.751	0.454
LVESD (mm)	33.88 ± 5.58		34.27 ± 4.35		0.256	0.798
LVEF	0.61 ± 0.04		0.58 ± 0.04		− 2.648	0.009**
TAD (mm)	34.05 ± 3.12		35.60 ± 4.10		1.723	0.088
Length of septal annulus (cm)	3.75 ± 0.38		4.15 ± 0.54		3.546	0.001**
Length of anterior annulus (cm)	4.47 ± 0.53		5.15 ± 0.57		4.619	0.001**
Length of posterior annulus (cm)	3.40 ± 0.48		3.91 ± 0.65		3.710	0.001**
TAC (cm)	11.62 ± 1.11		13.21 ± 1.35		5.028	0.001**
TACI (cm/m^2^)	6.77 ± 0.83		8.39 ± 0.61		7.259	0.001**

*P < 0.05; **P < 0.001 for Continuous variables analysis

### Multivariable analysis of risk factors of significant recurrent tricuspid regurgitation

It was revealed by multivariable analysis that TACI (OR: 8.815, 95% CI: 2.599–25.773, *p* = 0.001), Postoperative Af (OR: 3.645, 95% CI: 1.078–16.437, *p* = 0.043) were risk factors of recurrent tricuspid regurgitation. TACI and Postoperative Af were considered as the independent predictor of postoperative recurrent tricuspid regurgitation (Table 6).

**Table 6:** Multivariable analysis of risk factors of significant recurrent tricuspid regurgitation

Variables	β	SE	Wald	*P* value	OR (95% CI)
TACI	2.102	0.585	12.903	0.001	8.815 (2.599–25.773)
Postoperative Af	1.324	0.787	2.829	0.043	3.645 (1.078–16.437)
Constant	− 17.859	4.374	16.672	0.002	

**Fig. 4:**
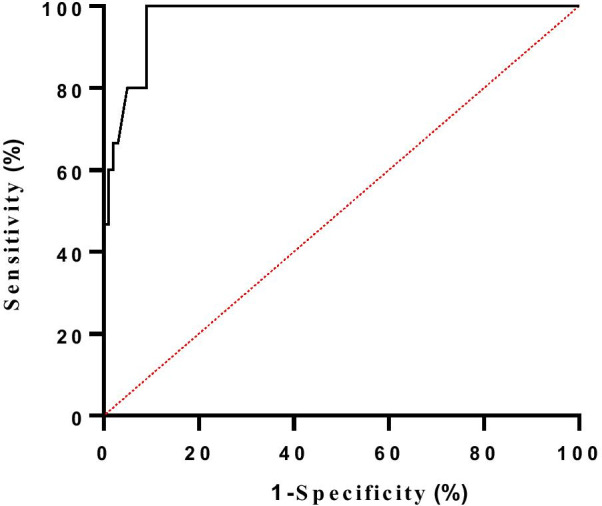
TACI of Receiver-operator curve (ROC) for predicting recurrent tricuspid regurgitation

Optimal cutoff was obtained through receiver operating characteristic curves analysis to predict recurrent tricuspid regurgitation. The ROC curves showed the optimal TACI cutoff was 7.86 cm/m^2^ (Table 7, Fig. 4).

**Table 7:** Sensitivity and Specificity of TACI as a risk factor to predict recurrent tricuspid regurgitation

Variables	Cut off point	Sensitivity %	Specificity %	AUC	*P* value
TAC/BSA	7.86 cm/m^2^	80.0	95.0	0.974	< 0.001

## Discussion

FTR is mainly caused by right atrial or right ventricular remodeling leading to tricuspid annulus dilation and leaflet tethering [6]. The anatomy of tricuspid valve is very complicated [7] and FTR is a kind of load-dependent disease. Volume load is positively associated with severity of tricuspid regurgitation. Moreover, it is often underestimated real severity of tricuspid regurgitation due to interference from diuretics [2]. However, accurate evaluation to severity of tricuspid regurgitation plays a critical role in clinical decision-making. Thus, the urgent demand seeking a reasonable method with the aim of assessing the severity of tricuspid regurgitation is the primary concentration in clinical practice. Rebecca T. Hahn [7] recommended vena contracta (VC) and effective regurgitant orifice area (EROA) were used to evaluate the severity of FTR, if VC ≥ 7 mm and EROA ≥ 40 mm^2^, severe FTR was confirmed. Recently, many attempts for instance, 2-d echocardiography, 3-d echocardiography, cardiac CT and MRI have been performed to assess the severity of FTR through tricuspid morphological changes, including the area and circumference of tricuspid annulus, the leaflet tethering distance and area, etc. [8]. Addetia et al. [9] used three-dimensional echocardiography to observe dynamic changes of tricuspid annular parameters during cardiac cycle. Then, the parameters including tricuspid annulus perimeter and area were measured by custom software and commercial multiplanar reconstruction method. If tethering distance > 8 mm and tethering area > 1.6 cm^2^, it can be defined that tethering extent of tricuspid leaflets was significant [10]. It has been shown that in FTR group, tricuspid annulus is more dilated and less nonplanar compared with control group without regurgitation, and the right ventricle and leaflet restriction are also different significantly in two groups. Hence, it has been supposed that morphological changes are effective and ideal in FTR evaluation.

### The association between the size of tricuspid annulus and the severity of regurgitation

In accordance with current European and American guidelines, surgical indications of FTR are recommended only based on the size of annulus measured by single 2-dimensional echocardiography under apical four-chamber view [11]. Obviously, this is not comprehensive and more accurate and objective parameters are required. Besides diameter of tricuspid annulus, both area and circumference may be valuable in evaluating tricuspid annular change. Mahia et al. [12], used three-dimensional echocardiography and commercial software to assess association between the tricuspid annular area and the severity of tricuspid regurgitation. Optimal cutoffs of tricuspid annulus dilatation were identified (3DA:10.4 cm^2^, 6.5 cm^2^/m^2^). Addetia et al. [9], have tried to use three-dimensional echocardiography to observe tricuspid valve geometry as well as dynamic changes and preliminarily quantify the tricuspid valve parameters. Nevertheless, these findings are not involved in guidelines yet, even though better results are presented. Otherwise, obtained data information is collected based on Western model. Special Chinese data is still lacking. Under this prerequisite, the feasibility of assessment focusing on the association between the TAC and FTR is investigated. Generally, measurement of TAC is processed by 3-d transthoracic echocardiography or transesophageal echocardiography which is more comprehensive in evaluating tricuspid morphological changes. However, all these mentioned methods are cumbersome and special tools must be prepared which means it is difficult to perform measurements in clinical practice. Interestingly, in our study, a simple, repeatable and practical method with a single silk thread was used. The one of main findings in our study is that there is close association between TAC and severity of FTR. Both TAC > 11.45 cm and TACI > 7.07 cm/m^2^ are considered as significant factors of tricuspid annulus dilatation for patients with FTR.

FTR will be worse if dilation of the tricuspid annulus progresses persistently. It has been indicated that during follow up, severity of FTR can be progressed from mild to severe. However, concomitant prophylactic tricuspid annuloplasty during mitral valve surgery is effective in decreasing recurrence of significant FTR at 5-year of follow up [13]. Sakata et al. [5] have also supported that concomitant tricuspid annuloplasty during left heart valve surgery benefits right ventricular geometry. So, tricuspid annuloplasty is recommended urgently and aggressively as excellent surgical strategy against FTR [14].

### Association between tricuspid annuloplasty and postoperative outcomes

Surgical procedures of tricuspid annuloplasty varies which depends on severity of regurgitation and surgeons’ experience. A consensus has been reached that prosthetic ring is the preferred option with better postoperative outcome. While, the outcome of flexible or rigid rings is unclear. Nishi et al. [15] indicated that postoperative motion and shape of annulus depended on the special type of prosthetic rings. It has been demonstrated that both mid-term and long term outcome of Edwards and Medtronic prosthetic rings are positive and encouraged [16]. Although, occasionally, adverse events including III degree atrioventricular block and suture dehiscence after tricuspid annuloplasty were reported [17], mostly, concomitant tricuspid annuloplasty is considered as a safe and effective procedure without increasing surgical time and incidence of postoperative complications. Rigid rings were selected in our study, and only 1 patient with postoperative III degree atrioventricular block received implantation of permanent pacemaker. Among remaining patients, no other prosthetic ring related complications were found. Itzhaki Ben Zadok et al. [18] also have demonstrated that concomitant tricuspid annuloplasty is potential and optimal in assessment of long term outcome for patients with rheumatic mitral valve disease.

### Risk factors of the recurrence of significant tricuspid regurgitation

It has been shown that a few risk factors of the recurrence of significant tricuspid regurgitation after tricuspid annuloplasty, such as severity of tricuspid regurgitation, size of right atrium or ventricle, size of left atrium or ventricle, atrial fibrillation, heart function, permanent pacemaker, leaflet tethering, recurrent mitral regurgitation and pulmonary hypertension [19, 20].

The current study showed that during longer than 1 year of follow up, there were 15 patients presented significant tricuspid regurgitation, which suggested that tricuspid recurrent regurgitation was associated with preoperative tricuspid annular circumference dilation (TACI > 7.86 cm/m^2^). Possibly, there are 3 reasons: (1) Dilated tricuspid annulus may accompanied with severe damage of tricuspid valve lesion and low right heart function which attribute the presence of recurrent regurgitation. (2) Once tricuspid annulus is dilated, right ventricular remodeling will occur. After tricuspid annuloplasty, original tricuspid valve mismatches altered annulus due to heart remodeling which leads to possible regurgitation. (3) During procedure of tricuspid annuloplasty, the initial stitch is generally targeted at the middle point of septal annulus. Larger and longer tricuspid annulus is not covered by ring completely which also leads to recurrent regurgitation. So, dilatation of tricuspid annulus plays an important role in recurrence of FTR and furthermore, TACI (OR 8.815, 95% CI 2.599–25.773, *p* = 0.001) is an independent predictor of postoperative recurrent tricuspid regurgitation. In our center, several studies also have been conducted with the aim of exploring the association between tricuspid annular circumference and postoperative tricuspid regurgitation and as a result, there is a close association between the circumference and tricuspid regurgitation. Zhu et al. [21] have recommended that TACI of 83 mm/m^2^ as the threshold of prophylactic tricuspid annuloplasty. Similarly, based on Mohammad Sharif Popal’s recommendation, the threshold is set as 80.2 mm/m^2^ [22]. Although no clear association between the size of ring and postoperative recurrent regurgitation is found in current study, we also observe that the possibility of postoperative regurgitation is greater after implantation of regular rings with large size. Meanwhile, an undersized (size 26 or 28) rigid ring has also been proved to be a potential option for treatment of FTR [23]. At the same time, in this study, it has been demonstrated that postoperative AF (OR 3.645, 95% CI 1.078–16.437, *p* = 0.043) is also a risk factor of recurrence of regurgitation. Positive association between atrial fibrillation and tricuspid regurgitation is found. From previous clinical experience and literature, ablation of atrial fibrillation is recommended strongly, not only for patients with small left atrium but for patients with large left atrium, which maintains perioperative sinus rhythm and benefits hemodynamic management. Because of the adverse effect of atrial fibrillation on tricuspid regurgitation, tricuspid annuloplasty was performed in all patients with preoperative atrial fibrillation, regardless of severity of regurgitation and annular size.

### Limitations and future investigations

This study only showed the risk factors of recurrent tricuspid regurgitation, but did not investigate the influence of recurrent tricuspid regurgitation on right heart function or survival rate, and did not provide any strategies dealing with these problems neither. So longer follow up data of these patients is still needed and handled. The sample of patients was relative small and some bias was inevitable. In future, the association between the severity of dilation from local lesions of tricuspid annulus and recurrent regurgitation should be emphazised.

## Conclusions

The severity of secondary tricuspid regurgitation is associated with the tricuspid annular circumference. The threshold cutoffs indicating significant tricuspid regurgitation (moderate and severe) are TAC 11.45 cm and TACI 7.07 cm/m^2^, respectively. Concomitant tricuspid annuloplasty is a safe and recommended procedure. Risk factors of significant recurrent tricuspid regurgitation after tricuspid annuloplasty during left heart surgery are significant tricuspid annulus circumference index > 7.86 cm/m^2^ and postoperative atrial fibrillation.

## Supplementary Information

**Additional file 1.** The availability of raw data and analysis.

## Data Availability

All data analysed during this study and its supplementary information files have been presented as separate ones of attached Excel Format.

## Abbreviations

FTR: Functional tricuspid regurgitation
TAC: Tricuspid annular circumference
TACI: Tricuspid annular circumference/body surface area
Af: Atrial fibrillation
MVP: Mitral vavle repair
MVR: Mitral vavle replacement
AVR: Aortic valve replacement
ASD: Atrial septal defect
CABG: Coronary artery bypass grafting
BSA: Body surface area
RA: Right atrium
RV: Right ventricle
LA: Left atrium
LVEDD: Left ventricular end‐diastolic diameter
LVESD: Left ventricular end‐systolic diameter
LVEF: Left ventricular ejection fraction
TAPSE: Tricuspid annular plane systolic excursion
PASP: Pulmonary arterial systolic pressure
A4C D: Tricuspid annular dimension in apical four-chamber view
NYHA: New York Heart Association
